# Efficacy Remaining at Time of Requested Re-Treatment for Cervical Dystonia: A Potential New Treatment Paradigm with DaxibotulinumtoxinA

**DOI:** 10.3390/toxins17030133

**Published:** 2025-03-12

**Authors:** Aaron Ellenbogen, Robert A. Hauser, Atul T. Patel, Peter McAllister, Todd M. Gross, Rashid Kazerooni, Conor J. Gallagher, David A. Hollander

**Affiliations:** 1 Quest Research Institute, 28555 Orchard Lake Road, Suite 200, Farmington Hills, MI 48334, USA; 2 Department of Neurology, University of South Florida, Tampa, FL 33613, USA; rhauser@usf.edu; 3 Kansas City Bone & Joint Clinic, Overland Park, KS 66211, USA; apatel@kcbj.com; 4 New England Institute for Neurology and Headache, Stamford, CT 06905, USA; peter@neinh.com; 5 Revance Therapeutics Inc., Nashville, TN 37203, USA; tgross@revance.com (T.M.G.); rashid.kazerooni@revance.com (R.K.); cgallagher@revance.com (C.J.G.); david.hollander@revance.com (D.A.H.)

**Keywords:** botulinum toxin type A, cervical dystonia, daxibotulinumtoxinA, dysphagia, dystonic disorders, muscle weakness, peptides, spasmodic torticollis, treatment

## Abstract

The therapeutic efficacy remaining from prior treatments with botulinum toxins (BoNTs) when cervical dystonia (CD) patients prefer to be re-treated has not been well characterized. Here, we assessed the residual therapeutic efficacy of BoNT injections at the time of a patient-desired re-treatment. In pivotal trials for daxibotulinumtoxinA (DAXI) in CD, subjects could request re-treatment before returning to pre-treatment symptom levels (defined as ≤20% of peak efficacy remaining). In this post hoc analysis of the Phase 3 ASPEN-OLS trial, the median percent efficacy remaining (based on change in TWSTRS total score) was determined in subjects who requested re-injection before returning to pre-treatment symptoms. Dysphagia and muscle weakness were evaluated in patients requesting re-treatment with efficacy remaining, relative to those waiting to return to baseline. There were 264 (28.7% of 920 total treatments) patient requests for re-treatment before returning to pre-treatment status across the study. The median percent efficacy remaining at the time of requested re-injection was 45.5%, which corresponded to a median of 16.0 weeks (range 10.9–40.3) post-treatment. The rates of dysphagia (≤4.9%) and muscle weakness (≤6.8%) were low and were not significantly different in those who waited for return to pre-treatment symptom status versus subjects who requested re-injection with efficacy remaining. A significant proportion of CD patients wished to be re-treated with efficacy still remaining from prior BoNT injections as early symptoms re-emerged. With the overall clinical profile of DAXI, physicians can safely provide individualized treatment regimens based on the treatment goals or symptomatic needs of their patients.

## 1. Introduction

Cervical dystonia (CD), or spasmodic torticollis, is a chronic condition characterized by involuntary contractions of neck muscles and is often associated with discomfort or pain [1,2,3,4,5]. The treatment of choice for the management of pain and other symptoms is repeated local injections of botulinum toxin (BoNT) [6,7,8,9,10]. However, therapeutic efficacy often decreases 8–10 weeks following treatment [11,12], which leads to symptom re-emergence between injections, producing a “rollercoaster” effect [12]. Due to BoNT product labeling by the United States Food and Drug Administration (FDA), as well as reimbursement practices, the minimum treatment interval is typically limited to no sooner than 12 weeks for all currently approved BoNT products (which is often rounded up to 90 days by payers) [13,14,15,16].

In a prior open-label study, which permitted patients with CD to request re-treatment at their preferred timing, roughly half of the patients desired re-treatment prior to the standard 12-week re-treatment interval [17]. This potential gap between the minimum re-treatment interval and the duration of effective symptomatic control with conventional BoNT treatments leads to prolonged periods in which CD patients experience unwanted symptoms prior to their next treatment being administered and taking effect. Given that treating earlier than 12 weeks when symptoms begin to re-emerge with conventional BoNTs has not been a broadly viable option, the residual efficacy remaining from the previous injection cycle at the time of a patient-desired re-treatment has not been explored.

DaxibotulinumtoxinA (DAXI; DAXXIFY^®^, Revance Therapeutics, Nashville, TN, USA) is a recently approved BoNT type A product with a unique formulation that contains a custom-engineered 35-amino acid peptide (RTP004), and it has shown an extended clinical duration compared with conventional BoNTs, with a median duration of effect (based on time to loss of 80% of peak effect) of 20–24 weeks [18]. DAXI is also formulated without human serum albumin and manufactured without accessory proteins [19]. The highly positively charged RTP004 peptide binds noncovalently to the negatively charged surfaces of the 150 kDa core neurotoxin, increasing neuronal cell binding affinity and SNAP-25 cleavage in a dose-dependent manner [19,20,21]. Additionally, in both pre-clinical [22] and clinical studies [18,23], DAXI has demonstrated wide safety margins. This is thought to result from low quantities of active neurotoxin, due to the greater bioavailability of the toxin at the nerve terminal, as well as the potential role of the RTP004 peptide in reducing the diffusion of BoNT away from the injection site to off-target tissues [18,20].

The double-blind, placebo-controlled Phase 3 (ASPEN-1) [18] and open-label extension (ASPEN-OLS) [23] trials for DAXI in CD allowed subjects to request re-treatment prior to returning to pre-treatment symptom levels. The objective of this post hoc analysis of the Phase 3 ASPEN-OLS study was to determine the residual therapeutic efficacy of prior BoNT injection at the time of a patient-desired re-treatment.

## 2. Results

### 2.1. Baseline Characteristics

In ASPEN-OLS, a total of 357 subjects received at least one re-treatment and were included in the analysis. Approximately half of the subjects (*n* = 177, 49.6%) in the study requested re-treatment at least once before returning to baseline symptom status. There were no significant differences in baseline characteristics between those who waited to return to baseline and those who requested re-treatment (Table 1), except that (1) male subjects were significantly more likely than females (64.7%, 77 of 119 vs. 42.0%, 100 of 238; *p* = 0.0001) to request early re-treatment before returning to baseline and (2) patients requesting re-treatment reported a 1-point higher baseline TWSTRS pain score than did those who always returned to baseline (10.8 vs. 9.8, *p* = 0.013).

### 2.2. Percent Efficacy Remaining at Re-Treatment Request

When re-treatment was requested prior to returning to baseline, the median percent of peak efficacy (based on change from peak Toronto Western Spasmodic Torticollis Rating Scale, TWSTRS) remaining at the time of the request was 45.5%, corresponding to a median of 16.0 weeks post-treatment based on Cycle 1 and Cycle 2. The median efficacy remaining was 41.6% in Cycle 1 (116 requests, Table 2) and 49.2% in Cycle 2 (107 requests). Similar percent efficacy remaining was observed for the three TWSTRS subscales of pain (55.6%), disability (50.0%), and severity (40.8%), across Cycles 1 and 2. The requests for re-treatment corresponded to a median re-treatment interval of 17.0 (range 11.4–40.3) weeks in Cycle 1 and 15.3 (range 10.9–28.1) weeks in Cycle 2. Additional cycles were not included in this analysis because Cycles 3 and 4 were artificially truncated based on the study protocol.

Based on Kaplan–Meier analysis, the overall median time to loss of 50% peak efficacy among all the DAXI-treated patients (*n* = 357) was 16.7 weeks. For Cycle 1, the time taken to reach a loss of 50% peak efficacy among all the DAXI-treated patients was 16.6 weeks, and for Cycle 2, it was 17.1 weeks.

### 2.3. Re-Treatment Characteristics

The peak symptomatic improvement across all cycles in ASPEN-OLS, as measured by the mean reduction in TWSTRS total score at Weeks 4 and 6, was similar between those who requested re-treatment prior to returning to baseline (−18.4) versus those who waited to return to baseline symptom status (−17.8).

Those patients who requested re-treatment with efficacy remaining were less likely to have had their doses titrated from prior treatments than those who waited to return to baseline (35.2%, 93 of 264, titrated vs. 43.4%, 139 of 320, respectively; *p* < 0.05). In addition, the mean dose titration across cycles was also significantly lower for those who requested re-treatment prior to returning to baseline (12.3U, SD = 30.8 vs. 22.7U, SD = 31.2; *p* < 0.0001). 

### 2.4. Safety

The rates of dysphagia and muscle weakness remained low throughout the study. There were no statistically significant differences between the rates of dysphagia (4.9%, 3.8%, *p* = 0.5409) or muscle weakness (6.8%, 4.4%, *p* = 0.2603) depending on whether patients were re-treated with efficacy remaining from their prior DAXI treatment or patients chose to return to baseline before being re-treated, respectively (Table 3).

## 3. Discussion

These findings not only confirm that many CD patients prefer to be re-treated with BoNTs prior to returning to their baseline level of symptoms but also demonstrate that many patients seek re-treatment with approximately 40–50% of the efficacy remaining from their previous treatment cycle. This is the first analysis to report patient requests for re-treatment in the context of residual efficacy remaining from the previous injection cycle.

There is prior evidence establishing that many patients desire re-treatment prior to 12 weeks and are often dissatisfied with the traditional 12-week treatment paradigm with conventional BoNTs. In a previous phase 3 flexible-interval open-label study with incobotulinumtoxinA, where subjects requested re-treatment injection intervals, nearly half of the requested cycles were less than every 12 weeks, with 22.5% requesting re-injection within 6–10 weeks [17]. A patient survey in CD has also shown that two-thirds of patients do not achieve 3 months of adequate symptom relief with conventional BoNTs [12]. A separate survey showed similar findings, with a 9.5-week mean decline in conventional BoNT effect and 45% reporting a preferred treatment cycle of ≤10 weeks. Of note, the onabotulinumtoxinA prescription information for CD states that in their pivotal trial (which was enriched for responders), “the majority of patients who had shown a beneficial response by Week 6 had returned to their baseline status by 3 months after treatment”.

Our results demonstrate that the mean dose titration across cycles was significantly lower for patients requesting re-treatment prior to returning to baseline (12.3U, SD = 30.8 vs. 22.7U, SD = 31.2; *p* < 0.0001). This is likely due to the remaining efficacy from prior treatment; however, additional factors may include patients and physicians seeking to time re-treatment in such a way that diminishes the waning of symptom relief as efficacy decays and the subsequent gradual increase of effect following re-treatment. The calculation of percent efficacy remaining in patients making a request provides the first estimate in the literature of when patients chose to be re-treated. The time at which 50% efficacy is lost may represent an optimal re-treatment interval for some patients, though further study is warranted to assess the reasons for this choice.

While other studies have explored the duration of action as time to loss of 80% of efficacy remaining via a similar methodology to the present study [24], this is the first analysis to explore the percentage of peak efficacy remaining at the time of patient request for re-injection. Although ASPEN-OLS did not ask patients for their preferred threshold for re-injection, the results suggest that many would like to be re-injected by the time they have experienced approximately a 50% loss of effect, which may occur earlier for many patients with conventional BoNTs than the standard 12-week injection interval. In the current analysis, those who requested re-treatment before returning to baseline with approximately 50% efficacy remaining did so at approximately 16 weeks from their prior treatment. This interval was also consistent across the entire study, in which 50% loss of effect across all patients was observed between 16 and 17 weeks. Given that DAXI’s duration of action typically exceeds the standard 12-week minimum re-treatment interval, CD patients now have the opportunity to receive re-treatments at their preferred re-treatment intervals, whether this is at 12 weeks, with potentially greater symptomatic coverage, or longer. This new approach may optimize treatments to individual patient preferences, with future studies ideally aimed at evaluating the impact of timing the re-treatment on patient quality of life.

In this study, males were significantly more likely than females to request re-treatment prior to returning to baseline (Table 1), which may be related to gender differences in treatment benefit. Gender differences in patient care are reported for cervical dystonia, where males are less likely to experience an objective benefit with botulinum toxin treatment and more likely to cease treatment than females [25]. Additionally, males are likely to experience routine underdosing due to muscle mass, given a fixed dose.

The adverse event rates for DAXI remained low across both re-treatment scenarios, including when re-treatment took place with efficacy remaining from prior treatments. This is consistent with the safety findings in ASPEN-1 and ASPEN-OLS, which demonstrated lower rates of adverse events, particularly dysphagia and muscular weakness, relative to those previously reported in pivotal trials for other FDA-approved BoNTs for CD (dysphagia: 13–39%; muscle weakness: 7–16%) [13,17,26,27,28]. Furthermore, while neutralizing antibodies can have a clinical impact, the rare occurrence (1/357) in this study is unlikely to have influenced our conclusions. DAXI’s wide safety margins are thought to be a direct result of the novel DAXI formulation, which contains a custom-engineered peptide (RTP004). This highly positively charged peptide promotes electrostatic binding to negatively charged extracellular structures, such as neuronal surfaces and extracellular matrix proteins [21], thereby increasing neuronal bioavailability and reducing the amount of core neurotoxin necessary to achieve a desired effect relative to conventional BoNTs [29]. Additionally, the peptide is thought to minimize unwanted spread to off-target tissues [20,21], as demonstrated in an animal study comparing DAXI to onabotulinumtoxinA [30] and evidenced by low rates of dysphagia in pivotal clinical studies.

A key limitation of this post hoc analysis is that the originator studies were not designed to evaluate patient preferences for treatment thresholds or intervals in a real-world setting. As such, patients could be re-injected once they had returned to baseline, or possibly sooner, if requested and in agreement with their clinician. Because of this study’s design, patients may have waited longer than they might have had the protocol indicated that they were to be re-injected when they felt it most appropriate based on return of symptoms. This needs to be evaluated in a future study in CD patients, particularly in populations not included in the current study, such as those with predominant retrocollis or anterocollis postures. Further, subjects were not randomized to receive re-treatment, neither after a return to pre-treatment status nor following a request with efficacy remaining, such that individual subjects could have successive treatments under both scenarios. An additional limitation is that the ASPEN-OLS study was not designed specifically to provide an estimate of percent efficacy remaining at the time of desired re-treatment. The study patients who requested re-treatment prior to returning to baseline may have different psychological factors or motivations than those who waited to return to baseline. As such, re-treatment with 50% efficacy remaining may not be generalizable to all patients.

## 4. Conclusions

The findings of this post hoc analysis demonstrate that many CD patients prefer to be re-treated with a significant proportion of efficacy remaining from their prior BoNT treatments, consistent with early symptom re-emergence. With the extended duration of symptom relief provided by DAXI, there is a potential opportunity to provide better symptom control for some patients during a typical 12-week injection interval or the option of extending the time between treatments based on the patient and dose selection. This new approach may allow physicians the flexibility to optimize treatments to individual patient preferences.

## 5. Materials and Methods

### 5.1. Ethics Approval and Subject Consent

The study protocol received unconditional approval from the Copernicus Group Independent Review Board (CGIRB) on April 18, 2018. CGIRB coordinated the Institutional Review Board approval for each of the sites who participated in this study. Ethics approval was obtained by all study sites, and each study was conducted according to the principles of the Declaration of Helsinki and applicable laws and regulations. All subjects provided written informed consent. ASPEN-OLS (NCT03617367) is registered at www.clinicaltrials.gov and at ClinicalTrialsRegister.eu (EudraCT unique identifier: 2018-000447-11).

### 5.2. Study Design

ASPEN-OLS was a phase 3, open-label trial conducted at 64 sites in 9 countries in North America and the EU from August 2018 to May 2021 [23]. Adults (aged 18–80 years) with moderate-to-severe CD, defined as having a TWSTRS total score of ≥20, were eligible for inclusion. Subjects were excluded if they had CD with predominant retrocollis or anterocollis posture or CD that was attributable to an underlying etiology. Eligible subjects were those who completed the main phase of the ASPEN-1 trial or subjects who were naïve to BoNT treatment or had experience with BoNT treatment and demonstrated a response to their last BoNT treatment. Subjects were eligible for re-treatment if they reached their minimum residual therapeutic efficacy (loss of 80% of peak effect on change in TWSTRS total score), considered a return to pre-treatment symptom levels or earlier, upon patient request with investigator agreement.

Subjects were treated for up to 4 cycles with DAXI. Re-treatment was prohibited earlier than the 12-week timepoint (±14-day treatment window) and could not occur with less than 12 weeks remaining in the study. Subjects could receive a maximum of 4 treatments within the 52-week study. The initial dose (Cycle 1) was 250U for subjects who had previously received ≥190U of onabotulinumtoxinA or incobotulinumtoxinA or for those who would qualify for this dose based on investigator judgment. All other subjects received an initial dose of 125U. Beginning with Cycle 2, the dose could be titrated based on the subject’s response to the previous cycle of treatment. The four pre-defined dose levels were DAXI 125U, 200U, 250U, and 300U. The doses were increased or decreased by a maximum of one pre-defined dose step (i.e., 50U or 75U) in each cycle. Re-treatment was not allowed after Week 40 to allow for a minimum follow-up period of 12 weeks in the final treatment cycle.

**Figure 1:**
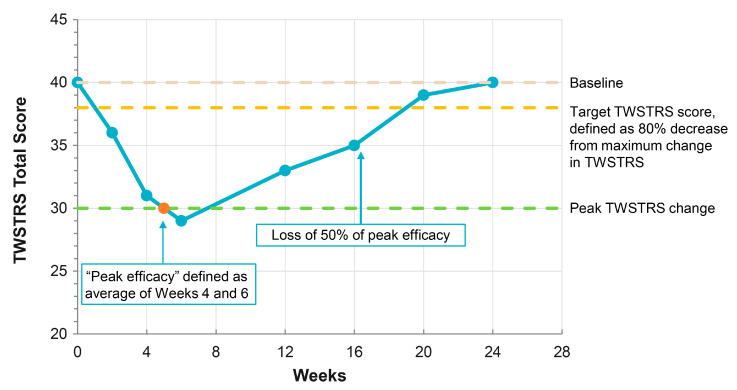
An example chart of the TWSTRS total score over time. The change from baseline in TWSTRS total score, averaged across Weeks 4 and 6, was the primary efficacy point (orange dot). The duration of effect was the time from treatment until loss of ≥80% of the peak treatment effect (i.e., average TWSTRS total score achieved at Weeks 4 and 6, shown as the orange dashed line). Abbreviation: TWSTRS—Toronto Western Spasmodic Torticollis Rating Scale.

The primary efficacy endpoint for the OLS study, as agreed upon with the FDA and the European Medicines Agency (EMA), was the change from baseline in TWSTRS total score averaged across Weeks 4 and 6 within each cycle (Figure 1, orange dot). The duration of effect was defined as the time from treatment until loss of ≥80% of the peak treatment effect (TWSTRS total score averaged across Weeks 4 and 6), consistent with prior studies [24,31], and was evaluated for treatment Cycles 1 and 2, which were not artificially truncated in this 52-week study (Figure 1, orange dashed line). Safety was evaluated at all study visits.

### 5.3. Statistical Analysis

All 357 subjects enrolled in ASPEN-OLS were included in this post hoc analysis. Each treatment in Cycles 1, 2, and 3 (920 treatments) was categorized as (1) ending by reaching minimum residual benefit in TWSTRS (i.e., < 20% efficacy remaining/loss of 80% of peak effect), (2) ending in a request for re-treatment with >20% efficacy remaining, or (3) another outcome (e.g., reached end of study, etc.). Demographic and baseline characteristics were compared between the group of subjects who only had treatments that ended in reaching the target versus those with at least 1 request for re-treatment prior to reaching the target. Comparisons were performed using *t*-tests for quantitative variables (e.g., age) and chi-square or exact tests for qualitative variables (e.g., sex). The percentage of efficacy remaining at the end of each treatment was calculated as the change from baseline at the final visit for that treatment divided by the peak change from baseline and summarized (median) for treatments ending in a request. The duration of each treatment was calculated as weeks from treatment date to end of treatment and was summarized (median) separately for treatments ending in reaching the target and a request for re-treatment. Based on the median percent efficacy remaining, a Kaplan–Meier time to loss of 50% efficacy was performed using all treatments, and the median survival estimate was reported. The incidence rate of dysphagia and muscle weakness was calculated using the subset of treatments ending in reaching the target or requesting re-treatment that were followed by a subsequent re-treatment. Statistical analyses were undertaken using SAS version 9.4 (SAS Institute, Cary, NC, USA).

## Figures and Tables

**Table 1:** Demographics and baseline disease characteristics.

	≥1 Requests for Re-Treatment Prior to Returning to Baseline ^a^ (*n* = 177)	All Treatments After Return to Baseline Symptom Status ^b^ (*n* = 180)	*p*-Value
Sex, female	56.5%	76.7%	0.0001
Age, years, mean (SD)	57.2 (11.7)	58.0 (12.0)	0.4952
Baseline severity, TWSTRS total, mean (SD)	44.1 (10.3)	42.5 (9.8)	0.1307
Severity Subscale, mean (SD)	20.7 (3.9)	20.6 (4.0)	0.8187
Pain Subscale, mean (SD)	10.8 (4.1)	9.8 (3.8)	0.0113
Disability Subscale, mean (SD)	12.5 (5.0)	12.1 (5.3)	0.4049
Toxin experienced	85.3%	77.8%	0.0668
Received DAXI 125U in Cycle 1	26.6% ^b^	35.6% ^b^	0.0662

^a^ Requested re-treatment with >20% efficacy remaining ≥1 time during study; ^b^ waited until ≤20% efficacy remaining for all treatments. Abbreviations: DAXI—DaxibotulinumtoxinA; SD—standard deviation; TWSTRS—Toronto Western Spasmodic Torticollis Rating Scale; U—units.

**Table 2:** Treatments in ASPEN-OLS.

	*n*	Subject Requested Re-Treatment with Efficacy Remaining ^a^	Subject Treated After Return to Baseline Symptom Status ^b^	Other ^c^
Cycle 1	357	116 (32.5%)	206 (57.7%)	35 (9.8%)
Cycle 2	329	107 (32.5%)	120 (36.5%)	102 (31.0%)
Cycle 3	234	41 (17.5%)	47 (20.1%)	146 (62.4%)
All cycles	920	264 (28.7%)	373 (40.7%)	283 (30.7%)

^a^ Received re-treatment with ≥20% of peak efficacy remaining. ^b^ Treated after return to <20% of peak efficacy remaining. ^c^ Other outcomes include reached end of study, no improvement from baseline at Weeks 4 and 6, and early termination.

**Table 3:** Rates of dysphagia and muscle weakness by percent efficacy remaining.

	Requested Re-Treatment (≥20% Efficacy Remaining) *n* = 264 ^a^	Returned to Baseline (<20% Efficacy Remaining) *n* = 320 ^a^	*p*-Value
Dysphagia	13 (4.9%)	12 (3.8%)	0.5409
Muscle weakness	18 (6.8%)	14 (4.4%)	0.2063

^a^ *n* denotes the number of treatments.

## Data Availability

The original contributions presented in this study are included in the article. Further inquiries can be directed to the corresponding author.

## Abbreviations

The following abbreviations are used in this manuscript:

BoNT	Botulinum toxin
CD	Cervical dystonia
DAXI	DaxibotulinumtoxinA
FDA	Food and Drug Administration
SD	Standard deviation
TWSTRS	Toronto Western Spasmodic Torticollis Rating Scale
U	Units
